# Functional Connectivity of Red Chlorophylls in Cyanobacterial Photosystem I Revealed by Fluence-Dependent Transient Absorption

**DOI:** 10.1021/acs.jpcb.5c00198

**Published:** 2025-03-18

**Authors:** Sara H. Sohail, Siddhartha Sohoni, Po-Chieh Ting, Lexi R. Fantz, Sami M. Abdulhadi, Craig MacGregor-Chatwin, Andrew Hitchcock, C. Neil Hunter, Gregory S. Engel, Sara C. Massey

**Affiliations:** † Department of Chemistry, Institute for Biophysical Dynamics, the James Franck Institute, and the Pritzker School for Molecular Engineering, The University of Chicago, Chicago, Illinois 60637, United States; ‡ Laboratory of Chemical Physics, National Institute of Diabetes, and Digestive, and Kidney Diseases, National Institutes of Health, Bethesda, Maryland 20892, United States; § Department of Chemistry and Biochemistry, Swarthmore College, Swarthmore, Pennsylvania 19081, United States; ∥ Department of Chemistry and Biochemistry, Southwestern University, Georgetown, Texas 78626, United States; ⊥ School of Biosciences, University of Sheffield, Sheffield S10 2TN, U.K.

## Abstract

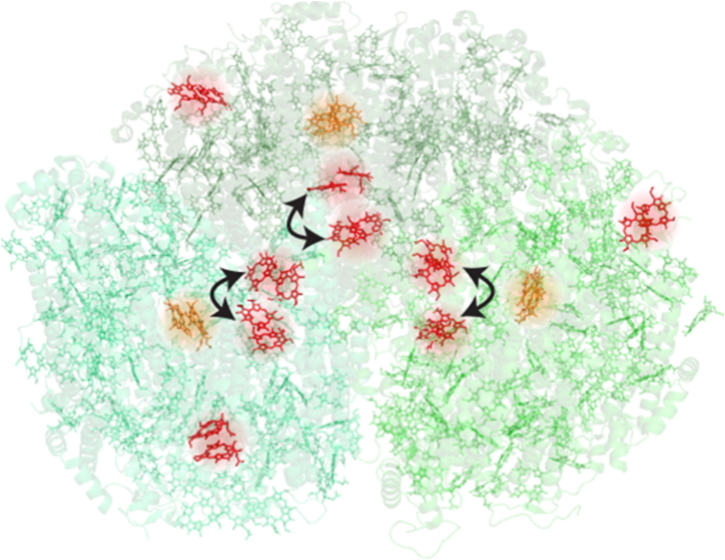

External stressors modulate the oligomerization state of photosystem I (PSI) in cyanobacteria. The number of red chlorophylls (Chls), pigments lower in energy than the P_700_ reaction center, depends on the oligomerization state of PSI. Here, we use ultrafast transient absorption spectroscopy to interrogate the effective connectivity of the red Chls in excitonic energy pathways in trimeric PSI in native thylakoid membranes of the model cyanobacterium *Synechocystis* sp. PCC 6803, including emergent dynamics, as red Chls increase in number and proximity. Fluence-dependent dynamics indicate singlet–singlet annihilation within energetically connected red Chl sites in the PSI antenna but not within bulk Chl sites on the picosecond time scale. These data support picosecond energy transfer between energetically connected red Chl sites as the physical basis of singlet–singlet annihilation. The time scale of this energy transfer is faster than predicted by Förster resonance energy transfer calculations, raising questions about the physical mechanism of the process. Our results indicate distinct strategies to steer excitations through the PSI antenna; the red Chls present a shallow reservoir that direct excitations away from P_700_, extending the time to trapping by the reaction center.

## Introduction

**Figure 1:**
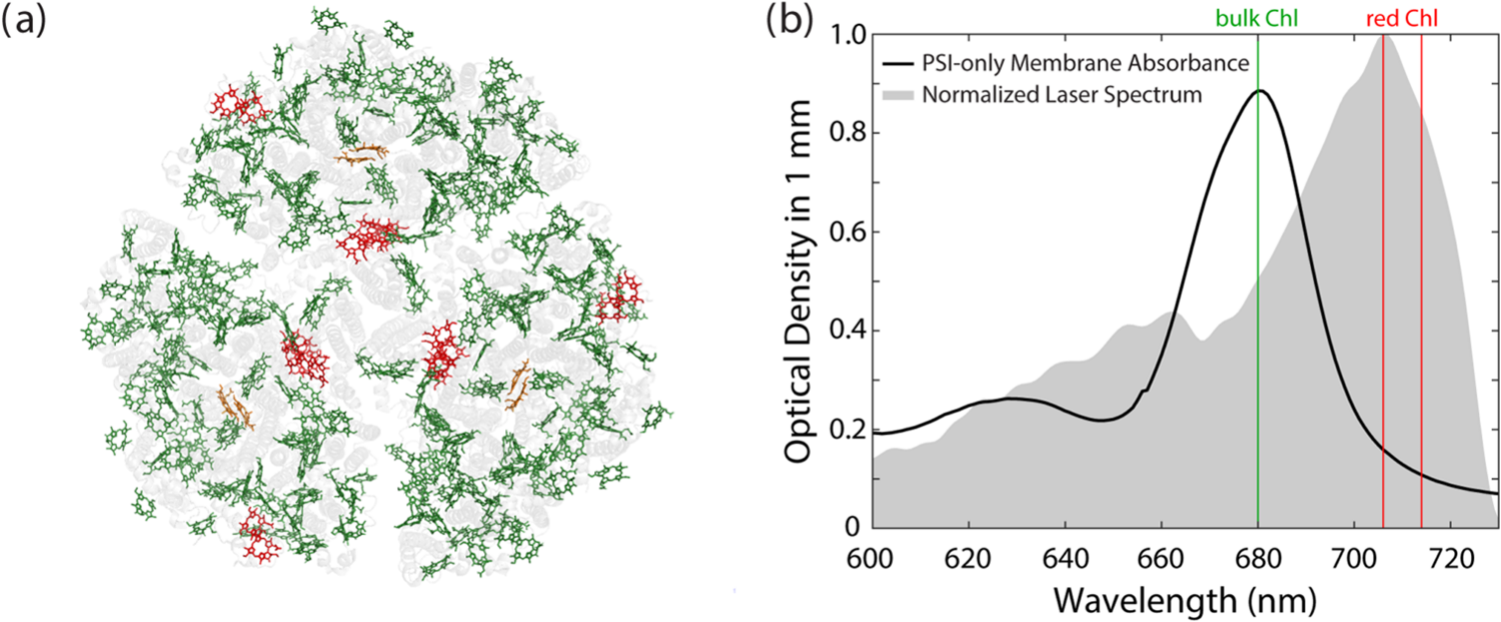
(a) Crystal structure of the PSI trimer from *Synechocystis* sp. PCC 6803 (protein structure shown based on data for PDB ID: 5OY0(30)). Bulk Chls are shown in green, and the red Chls are shown in red in the positions implicated by Akhtar et al.^14^ (A32, B7, B31, B32, B37, B38); the P_700_ special pair Chls are shown in orange. Chl phytol tails, carotenoids, phylloquinones, and Fe–S clusters have been removed for the sake of clarity. (b) Linear absorption spectrum (black) showing the optical density of PSI-only membranes in a 1 mm sample cell and the laser spectrum (gray) used for power-dependent transient absorption (TA) experiments. Green line indicates the spectral location of the absorption maximum for bulk Chls at 680 nm. Red lines indicate the previously reported spectral locations of the red Chls at 706 and 714 nm.^11,13,25^

Photosystem I (PSI), a fused antenna/reaction center (RC) complex, is integral to oxygenic photosynthesis, and in cyanobacteria, it is the more abundant photosystem complex.^1−6^ The structure of cyanobacterial PSI from *Synechocystis* sp. PCC 6803 is shown in Figure 1a. Despite modifications of the basic architecture between cyanobacteria, algae, and plants,^4,7,8^ the same light-harvesting processes are found: pigments in PSI directly absorb solar photons or accept excitations from other light-harvesting antenna complexes. Excitations are then transferred to a centrally located RC where charge separation occurs at a pair of specialized Chls, denoted P_700_.^5,9^ The majority of Chl *a* pigments in PSI, known as bulk Chls, have a peak *Q*_*y*_ absorption at 680 nm. Generally, excitations migrate through PSI bulk Chls until they reach the P_700_ RC (peak absorption at 700 nm). However, the antenna in cyanobacterial PSI contains a subset of pigments with absorption shifted to lower energy than P_700_, which represents an energy difference on the order of *kT* at room temperature.^10−12^ These red Chls offer an alternative downhill energetic pathway from the bulk antenna Chls with the potential to draw excitations away from the RC.^13,14^ The red Chls vary in number and energy between different species of cyanobacteria and between different oligomerization states (typically trimeric or monomeric) of PSI.^10,15−19^ Though energy transfer and charge separation in PSI have been studied for several decades,^10,20−24^ questions remain about the specific role of the red Chls in the PSI light-harvesting process. We investigate exciton migration through the red Chls to elucidate how this small population of sites can act as a shallow trap for excitations before migrating to the bulk Chls or P_700_. The red Chls are an intrinsic feature of PSI electronic structure offering an opportunity to mitigate excess excitations, should the need arise, pointing to the red Chls as essential components of excitonic energy pathways in PSI.

Many studies have focused on identifying the precise location of red Chl sites and their participation in energy transfer through PSI.^11−14,25−29^ Conclusive findings have been stymied by the small number of red Chl pigments (only ∼5 red Chls out of 95 Chl *a* molecules per monomer for the trimeric PSI of *Synechocystis* sp. PCC 6803),^14,30^ the spectral overlap of all of the Chl pigments, and the temporal overlap of the energy transfer dynamics within PSI. The recent determination of the crystal structure of PSI in *Synechocystis* sp. PCC 6803 by Malavath et al. has led to refined proposed assignments of red Chl pigments in this species,^14,25,30^ designating two distinct pools of red Chls with absorption at 706 and 714 nm.^11,13,25^ While the red Chls are slightly energetically distinct from the bulk pigments, energy transfer between the bulk Chls and P_700_, the red Chls and P_700_, the bulk Chls and the red Chls, and trapping by P_700_ all occur on the femto- to pico-second time scales.^10,13,20−22,31−35^Figure 1 shows the room temperature linear absorption spectrum of membrane-bound PSI, the spectral locations of the bulk and red Chls, and their corresponding positions within a trimer.

In this article, we investigate the energetic connectivity of the red Chls in cyanobacterial PSI light harvesting at physiological temperature in the native membrane environment. To probe exciton migration through the red Chls to understand how these few lower energy pigments can temporarily detour excitations before they migrate to the bulk or P_700_, we employ fluence-dependent transient absorption (TA) spectroscopy. Our fluence-dependent measurements interrogate exciton migration in PSI trimers using singlet–singlet annihilation as a proxy for energy transfer between isoenergetic sites. Analysis of singlet–singlet annihilation has previously been used to recover inter- and intracomplex energy transfer times between spectrally overlapped states in plant and bacterial light-harvesting complexes.^36−38^ Annihilation rates depend on excitation density, connectivity, and size of domains within the system.^9,39−41^ To preserve the native structural and energetic architecture of PSI trimers and to avoid the use of detergents to solubilize these complexes, we prepared thylakoid membranes of a *Synechocystis* sp. PCC 6803 mutant lacking PSII (Δ*psbB*).^42−44^ Thus, we analyze native PSI complexes analogous to those imaged by atomic force microscopy in thylakoids from a range of cyanobacteria.^6^

## Methods

### Transient Absorption Spectroscopy

A 5 kHz Ti:sapphire laser (Coherent Inc.) was focused through 2 m of argon gas at 15 psi above atmospheric pressure to achieve a broadband white light spectrum, which was compressed using a multiphoton intrapulse interference phase scan (MIIPS) pulse shaper (Biophotonics Inc.). Each laser pulse passes through a 90/10 beam splitter to generate the pump and probe pulses, respectively. A motorized delay stage (Aerotech) was used to encode the waiting time delay between pump and probe pulses, and a 2.5 kHz optical chopper was used to block the pump beam in alternate laser shots. The pump pulse was focused to a 0.0050 mm^2^ spot through a 500 μm quartz flow cell (Starna Cells Inc.). The pump pulse was attenuated to achieve pump fluences of 21, 39, 74, and 117 nJ/pulse. These fluences correspond to 8.1, 15.2, 28.8, and 45.7 excitations per PSI monomer, respectively, calculated using the molar absorptivity of P_700_ determined by Müh et al.^45^ Details of these calculations are included in the Supporting Information. The probe beam and copropagating third-order signal are focused into a spectrometer (Andor Shamrock 303i) and imaged on a charge coupled device (CCD) line scan camera (Teledyne Dalsa) for heterodyne detection.

### Preparation of PSI-Only Thylakoid Membranes

The PSI-only Δ*psbB* mutant of *Synechocystis* sp. PCC 6803 used in this study has been described previously.^43^ In this strain, around half of PSI monomers are present in trimeric form and half in monomeric form (a trimer to monomer ratio of 1:3).^46^ Liquid cultures were grown in BG-11 media supplemented with 5 mM glucose at 30 °C under ∼50 μmol of photons m^–2^ s^–1^ white light illumination on an orbital shaker. Cells were pelleted and resuspended in buffer containing 25 mM potassium phosphate, pH 7.4, 100 mM NaCl, and 10 mM MgCl_2_. Resuspended cells were mixed with the same volume of glass beads and broken by 8 rounds of 60 s of bead beating in a Mini-Beadbeater (BioSpec Products). Following centrifugation at 6000*g* at 4 °C for 10 min to pellet unbroken cells and glass beads, the supernatant was removed and centrifuged at 20 000*g* at 4 °C for 30 min to separate the membrane pellet from the soluble fraction. The supernatant was discarded and the membrane pellet was resuspended in the same buffer as above. Samples for TA spectroscopy experiments were prepared to an optical density of ∼0.44 at 680 nm in a 500 μM quartz sample cell.

## Results and Discussion

**Figure 2:**
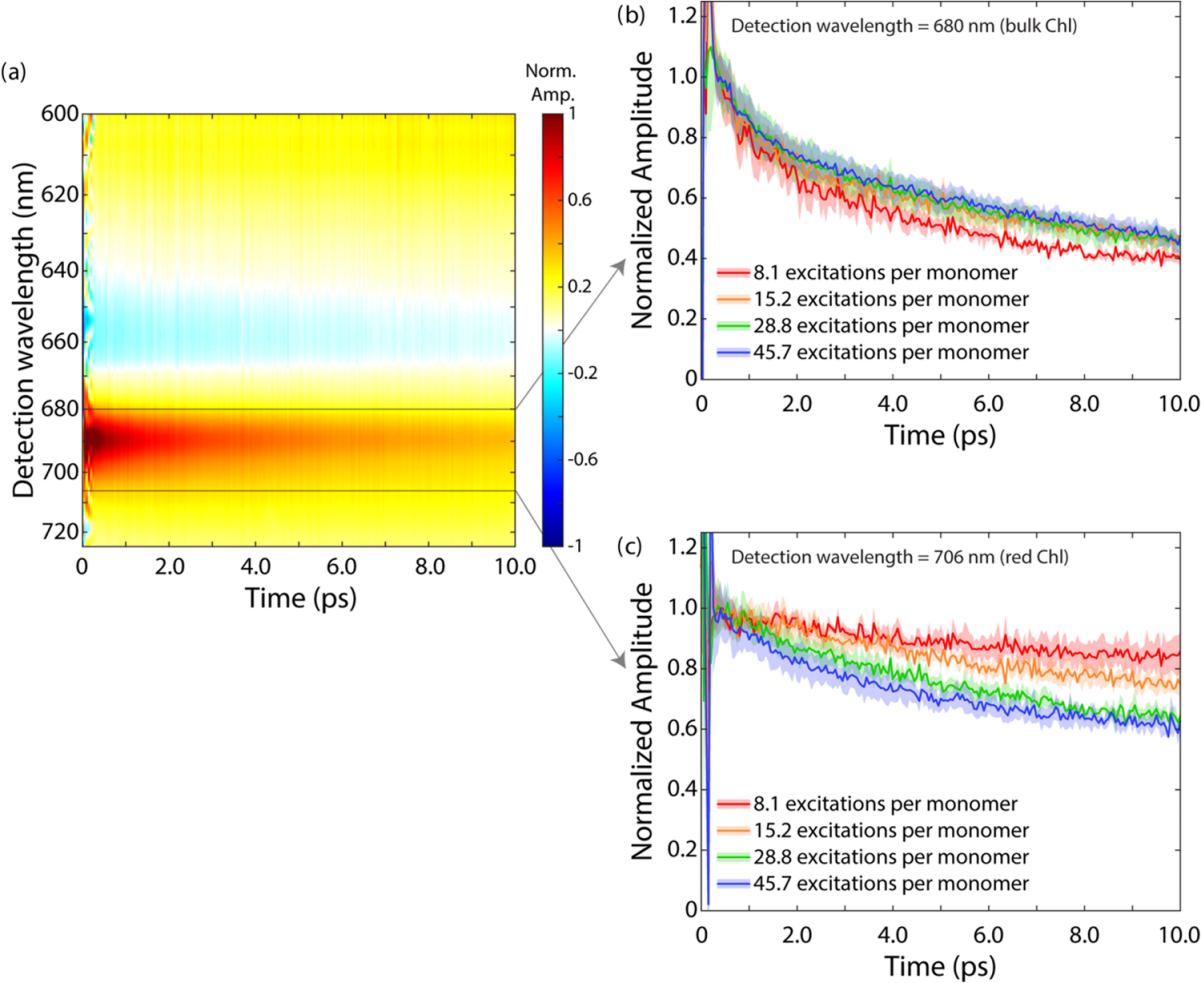
(a) TA spectrum of PSI-only membranes with an average fluence of 39 nJ/pulse, corresponding to 15.2 excitations per PSI monomer. The spectrum is an average of three runs, each normalized to the spectral maximum at 400 fs. Change in transmission is plotted: positive amplitude indicates SE/GSB signals, and negative amplitude indicates ESA signals. Waiting time traces from detection wavelengths of (b) 680 and (c) 706 nm at each fluence show dynamics from the spectral regions of the bulk Chls and red Chls, respectively. Each waiting time trace is an average of three runs, each individually normalized at 400 fs. Shaded error bars indicate the standard error of the mean.

PSI-only thylakoid membranes were excited with a broadband laser pulse (Figure 1b) and probed with the same laser spectrum. The laser spectrum (gray area in Figure 1b) was weighted to the redder wavelengths, ensuring that the red Chls were directly excited and probed. The TA spectrum at a pump power of 195 μW (39 nJ/pulse, 15.2 excitations per monomer) is shown in Figure 2a. Change in transmission is plotted: positive signals correspond to stimulated emission (SE)/ground state bleach (GSB) and negative signals indicate excited state absorption (ESA). We observe similar dynamics for each laser fluence at detection wavelengths of 680 (Figure 2b) and 685 nm (Figure S1b). These fluence-independent dynamics indicate a lack of annihilation in the bulk Chls on the time scale of our measurements. Data are normalized to the signal amplitude at 400 fs and we focus on dynamics at *T* ≥ 400 fs in this article; this conservative approach eliminates oscillations due to heterodyne signal from the scatter of the pump beam which adulterates early time signals. The bulk dynamics at each fluence fit well to a triexponential (Figure S2) with the intermediate and slow decay constants corresponding to transfer to P_700_ (16 ps)^13^ and a long-lived component (2 ns), respectively. The 2 ns time constant is in reasonable agreement with the 5 ns nondecaying component measured by Lee et al.^13^ The fast decay component can be fit to a ∼890 fs time constant, which agrees with previous subpicosecond measurements of equilibration and local energy transfer within the bulk Chls.^32,35^

The lack of fluence-dependent dynamics in the bulk Chls during the measured time window is noteworthy. Even at fluences of 45.7 excitations per monomer at which, on average, every 1 in 2 pigments is excited, we see no evidence of singlet–singlet annihilation in the bulk Chls on our resolvable time scale of 0.4–10 ps. Recent work has demonstrated that the annihilation rate is low (<0.1 per encounter) in materials when diffusion is fast,^47^ as it is through the bulk Chls.^22,23^ This expansive network of bulk Chls can support a large number of excitations for efficient light harvesting.

Though annihilation is absent in the bulk Chls, at longer wavelengths (Figure S1c–i), fluence-dependent dynamics emerge, showing faster decays of the SE/GSB signal at higher laser fluences. At 706 nm (Figure 2c), the spectral location of the red Chls, we observe an increase in the rate of decay as the average excitation density increases from 8.1 to 45.7 excitations per monomer. These fluence-dependent dynamics are consistent with an increased rate of singlet–singlet annihilation in the red Chls. As illustrated in the Jablonski Diagram in Figure S3b, one exciton relaxes to the ground state, and the other is promoted to a higher energy state before rapidly returning to the first excited state. The result is the annihilation of one of the original excitations. This process results in faster decay dynamics than are observed under conditions with lower excitation density (Figure S3a).^9,37^ Unlike in the bulk Chls where diffusion is fast,^22,23^ excitations have a longer dwell time at the small number of lower energy sites, increasing the rate of annihilation at the red Chls. Additionally, annihilation is greater in states with longer excited state lifetimes.^48^ As can be seen in the TA decays in Figure 2, bulk Chls have shorter excitation lifetimes than red Chl sites due to faster trapping by the P_700_ RC and downhill energy transfer into the red Chls, leading to more annihilation at the red Chls than at bulk Chls.

To connect the measured annihilation events with physical red Chl sites within PSI, we examine the assigned locations of the red Chl sites. Trimeric PSI, the most common oligomerization state, contain a greater number of red Chl pigments per monomer than monomeric PSI.^10,13,31,49,50^ Under typical growth conditions, the formation of trimeric PSI results in approximately three red Chl sites (two to three coupled Chls per site) per PSI monomer.^14,25^ Monomeric PSI contains approximately two red Chl sites; the Chls at one proximal site blue-shift in absorption and are no longer classified as red Chl pigments.^11,14,49^ Akhtar et al. assigned red Chl states to the A32/B7 and B31/B32 dimers with absorption at 706–707 nm, and the B37/B38 dimer with absorption at 714 nm.^14^ A31 is located between A32/B7 and B37/B38, couples to A32 (∼150 cm^–1^), and has been implicated as a red Chl by others.^16,23,51^ The B37/B38 and A32/B7 dimers are close to the contact point between monomers (proximal) in a PSI trimer, while the B31/B32 pair is located along the outer edge of the complex (distal), away from other monomers.^14,30^ The fast time scale of annihilation at the red Chls as illustrated by the dynamics at 706 nm presented in Figure 2, well within our 10 ps measurement window, points to the pair of proximal red Chl sites as the low energy trap for excitations.

To reconcile the observed fluence-dependent dynamics with a fundamental underlying physical mechanism, we calculated Förster resonance energy transfer (FRET) rates for excitation migration between red Chl sites and from red Chl sites to P_700_.^52,53^ Details of these calculations are included in the Supporting Information. The calculated FRET rate for transfer between proximal red Chl sites (A31/A32/B7 and B37/B38) is 16 ps, and the calculated rate between the distal dimer and either of the proximal sites is orders of magnitude slower (Table S1). However, in our experimental data, we observe robust picosecond hopping between red Chl sites, most likely the proximal red Chls. Our observed annihilation cannot be explained by a FRET-like hopping mechanism as the movement of energy between proximal red Chls happens much faster than would be expected by FRET rates. Picosecond time scales for energy transfer dynamics at the red Chls as fast 5–6 ps have been previously reported from cryogenic hole-burning and single-complex spectroscopy studies.^26,27^ At room temperature, our observed dynamics on the order of a couple of picoseconds would fit reasonably within existing data, but our data necessitate a mechanism beyond a dipolar free-space coupling.

**Figure 3:**
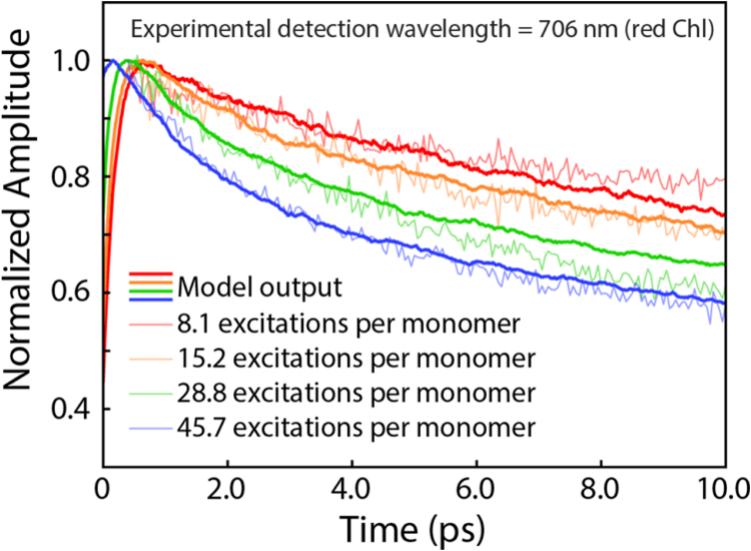
Overlay of modeled waiting time traces (bold lines) at each excitation density with experimental waiting time traces from the detection wavelength 706 nm (semitransparent lines). Simulation results are the average of 5000 iterations and are individually normalized to the maximum.

While our data are not consistent with a FRET-like energy transfer mechanism between the excitonic A31/A32/B7 and B37/B38 red Chl sites, we can reproduce the experimental dynamics using a diffusive random walk model (Supporting Information, Figures S6–S13) that allows the movement of excitations between proximal red Chl sites on a < 5 ps time scale. Details of the model are provided in the Supporting Information. Briefly, excitations are allowed to migrate between bulk and red Chl sites, with a probability determined by the time constant of the transfer between sites. As shown in Figure 3, we can successfully capture the picosecond annihilation dynamics at the red Chls, demonstrating that our experimental results are consistent with second-order kinetics, with the caveat that we do not have the theory to explain how excitons are moving so rapidly between proximal red Chl sites.

**Figure 4:**
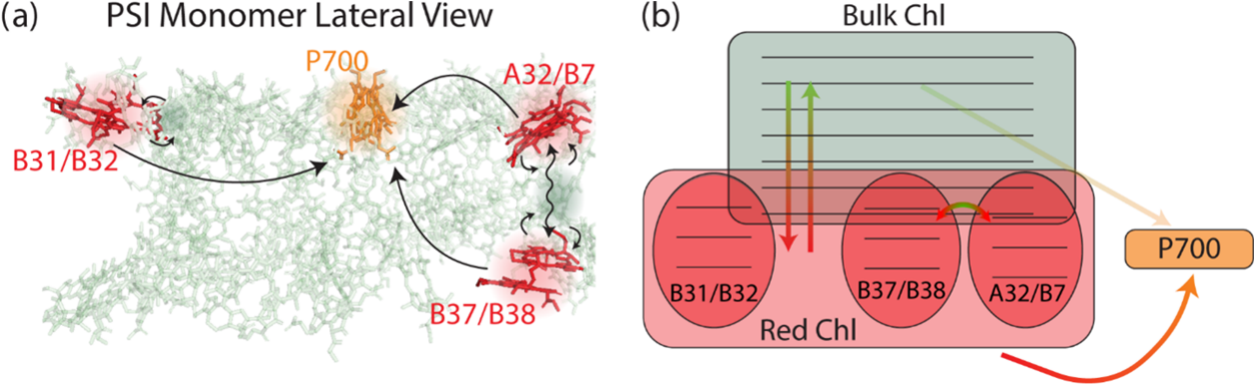
Proposed pathways of energetic motion involving the red Chls illustrated in (a) a lateral zoomed-in view of a PSI monomer and (b) a cartoon visualization using Chl locations from the crystal structure of the PSI trimer (protein structure shown based on data for PDB ID: 5OY0(30)) with red Chl assignments made by Akhtar et al.^14^ Energy moves between the two proximal red Chl sites (A32/B7 and B37/B38) within several picoseconds, necessitating more than free-space dipolar coupling. We hypothesize that low-lying bulk states mediate picosecond energy transfer between these red Chl sites.

The observed fluence-dependence at the red Chls is fully consistent with annihilation; however, this level of annihilation requires an unexpected physical parameter that is inconsistent with FRET-dynamics. The measured time scales of fluence-dependence point to a connectivity more sophisticated than simple dipole–dipole coupling, presumably mediated by bulk Chls. This requires us to include some bulk Chl states in a quantum mechanical sense. As shown in Figure 4, we hypothesize that there may be a subset of lower energy bulk Chls whose electronic states overlap with the excited states of the proximal red Chls, mediating transfer between the two red Chl sites. As simple models cannot explain our fluence-dependent TA data, more sophisticated QM/MM models may be needed to fully explain the mechanism of the energy flow between proximal red Chls. It is our hope that our data inspire further interrogation into the underlying physical mechanism giving rise to these surprisingly fast dynamics at the red Chls. The rapid energy transfer between these low energy sites is an intrinsic property of PSI, enabling the red Chls to act as a shallow reservoir for excitations, effectively increasing the transfer time to P_700_.

Even without a complete understanding of the physical mechanisms at play in exciton migration through the red Chls, our observed singlet–singlet annihilation requires a loss of excitations at the red Chl sites. Energy transfer from bulk to red sites could lead to annihilation at high excitation densities and a reduction in the total number of excitations in the system, but would not reduce the number of excitations at the red Chl sites and thus cannot fully explain our data. Our data require picosecond energy transfer between functionally connected proximal red Chl sites to reduce the number of excitations at these wavelengths, resulting in the observed fluence-dependent dynamics. These room temperature picosecond dynamics in intact thylakoid membranes are consistent with the earlier cryogenic measurements.^26,27^ The pair of proximal red Chl dimers serves as an intrinsic trap for excitations and increases the effective transfer time to reach the RC, which could be useful under specific environmental circumstances. As lone PSI monomers lack this pair of energetically connected proximal red Chls, this mechanism is unique to trimeric PSI. Our results support previous assertions that the red Chls act as an intrinsic trap that directs excitations away from the P_700_ RC, prolonging the time to trapping by P_700_.^31,54^

## Conclusions

Fluence-dependent ultrafast spectroscopy can elucidate the energetic connectivity between isoenergetic pigments. The role of cyanobacterial red Chls, which comprise a small number of pigments, in excitonic energy transfer in PSI has remained poorly understood due to spectral congestion. Transfer to P_700_ from the red Chls, while uphill, has an energy gap on the order of *kT* at room temperature suggesting that the red Chls offer a viable alternative pathway for excitations to move from the bulk Chls to P_700_.^14,54^ Our TA experiments show stark fluence-dependent dynamics due to singlet–singlet annihilation at the wavelengths corresponding to red Chls (∼706 nm). Our results support that the pair of proximal red Chl sites offer a shallow trap for excitations on their way to P_700_. While we do not yet fully understand the physics underlying the observed dynamics, we want to highlight the functional importance of energy transfer between red Chls on the picosecond time scale. This fast transfer enables the red Chls to act as an additional reservoir for excitations, effectively slowing some excitations on the way to P_700_. The lack of analogous fluence-dependence in the bulk Chl dynamics at an extremely high excitation density on the time scale of our experiments is notable and suggests that excitations move quickly through the bulk Chl to spatially separate excitations. The femtosecond bulk Chl dynamics at high excitation densities is an interesting avenue for further studies. Our results on intact cyanobacterial thylakoids indicate that the red Chls have an active and dynamic role in the excitonic pathways in cyanobacterial PSI and pave the way for further ultrafast and computational studies on the pathways of intra- and intermonomer PSI energy transfer.

## Data Availability

All data are included in the manuscript and the Supporting Information.

## Supporting Information Available

Additional TA waiting time data; triexponential fit to bulk Chl dynamics; illustration of energy transfer under high and low excitation densities; deviation of monoexponential fits at high and low fluence; FRET calculations; calculation of excitation densities; diffusive random walk model (PDF)
