# Supplementary material for: Functional Connectivity of Red Chlorophylls in Cyanobacterial Photosystem I Revealed by Fluence-Dependent Transient Absorption
Source: J Phys Chem B. 2025 Mar 18;129(12):3191–7. doi: 10.1021/acs.jpcb.5c00198 (PMC11956136; doi:10.1021/acs.jpcb.5c00198)
Supplement: Supplementary file 1 — jp5c00198_si_001.pdf [file jp5c00198_si_001.pdf]

Supporting Information: Functional Connectivity of Red Chlorophylls in Cyanobacterial Photosystem I Revealed by Fluence-Dependent Transient Absorption

Sara H. Sohail[1,2], Siddhartha Sohoni[1], Po-Chieh Ting[1], Lexi R. Fantz[3], Sami M. Abdulhadi[1], Craig MacGregor-Chatwin[4], Andrew Hitchcock[4], C. Neil Hunter[4], Gregory S. Engel[1], and Sara C. Massey[3*]

1. Department of Chemistry, Institute for Biophysical Dynamics, the James Franck Institute, and the Pritzker School for Molecular Engineering, The University of Chicago, Chicago, IL 60637

2. Laboratory of Chemical Physics, National Institute of Diabetes, and Digestive, and Kidney Diseases, National Institutes of Health, Bethesda, MD 20892; Department of Chemistry and Biochemistry, Swarthmore College, Swarthmore, PA 19081

3. Department of Chemistry and Biochemistry, Southwestern University, Georgetown, TX 78626

4. School of Biosciences, University of Sheffield, Sheffield S10 2TN, UK

*Corresponding Author E-mail and phone: masseys@southwestern.edu (512-863-1673)

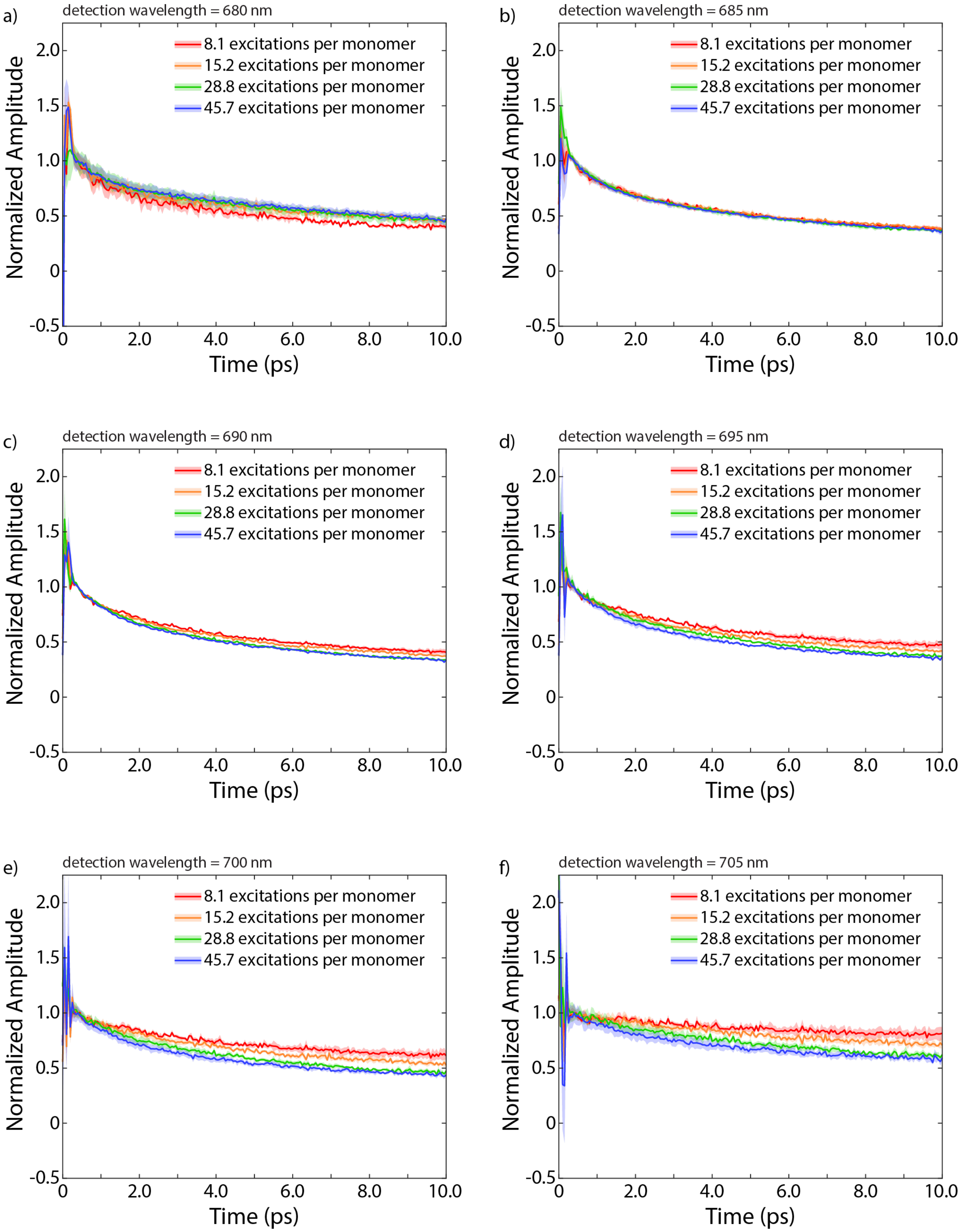
a)
detection wavelength = 680 nm
b)
detection wavelength = 685 nm
c)
detection wavelength = 690 nm
d)
detection wavelength = 695 nm
e)
detection wavelength = 700 nm
f)
detection wavelength = 705 nm
8.1 excitations per monomer
15.2 excitations per monomer
28.8 excitations per monomer
45.7 excitations per monomer
Normalized Amplitude
-0.5
0
0.5
1.0
1.5
2.0
Time (ps)
0
2.0
4.0
6.0
8.0
10.0

g)

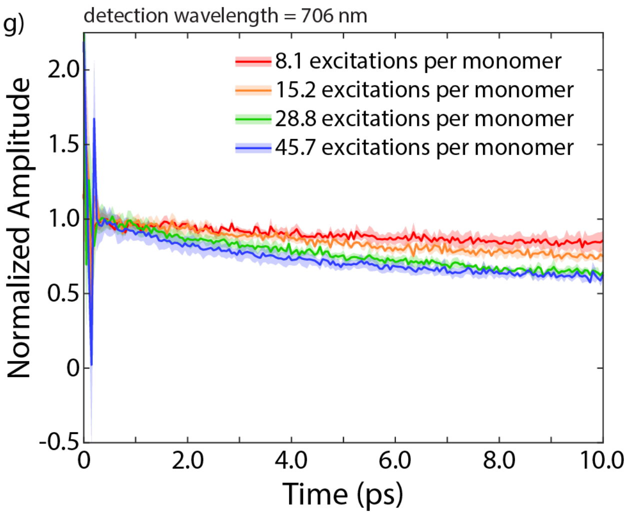

h)

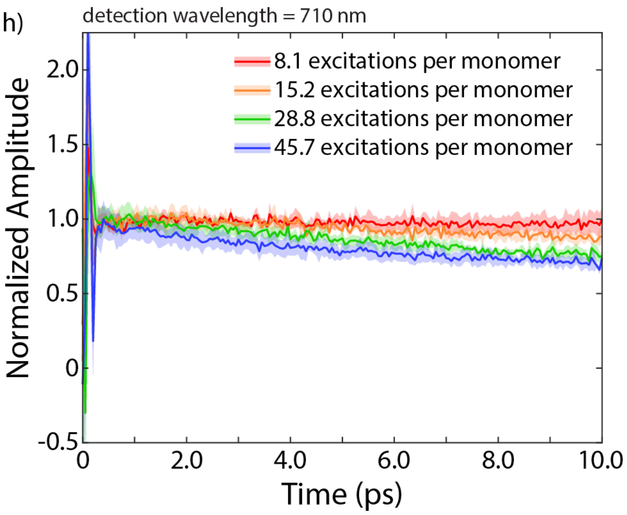

i)

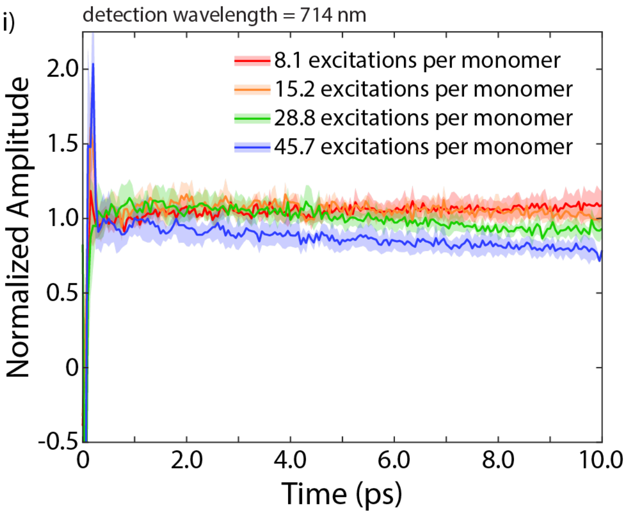

Figure S1. Waiting time traces from detection wavelengths ranging from a) 680 nm to i) 714 nm at each fluence. Each waiting time trace is an average of 3 runs, each individually normalized at 400 fs. Shaded error bars indicate the standard error of the mean.

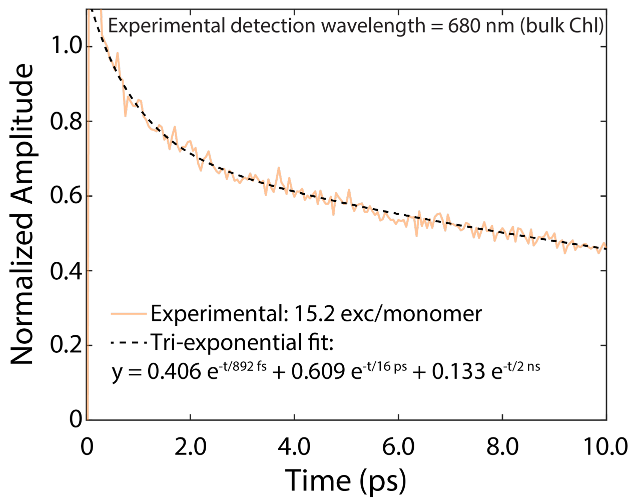

Figure S2. Waiting time trace from detection wavelength 680 nm (bulk Chl) at a pump fluence of 39 nJ/pulse with a tri-exponential fit to the waiting time trace. The data were fit with a fixed intermediate time constant of 16 ps corresponding to energy transfer from the bulk to P_{700} (time constant attributed to Lee *et al.*[1]) and a slow time constant of 2 ns. The amplitudes and the fast time constant were allowed to vary, and the best fit gives an 892 fs time constant consistent with equilibration and local energy transfer within the bulk Chl.

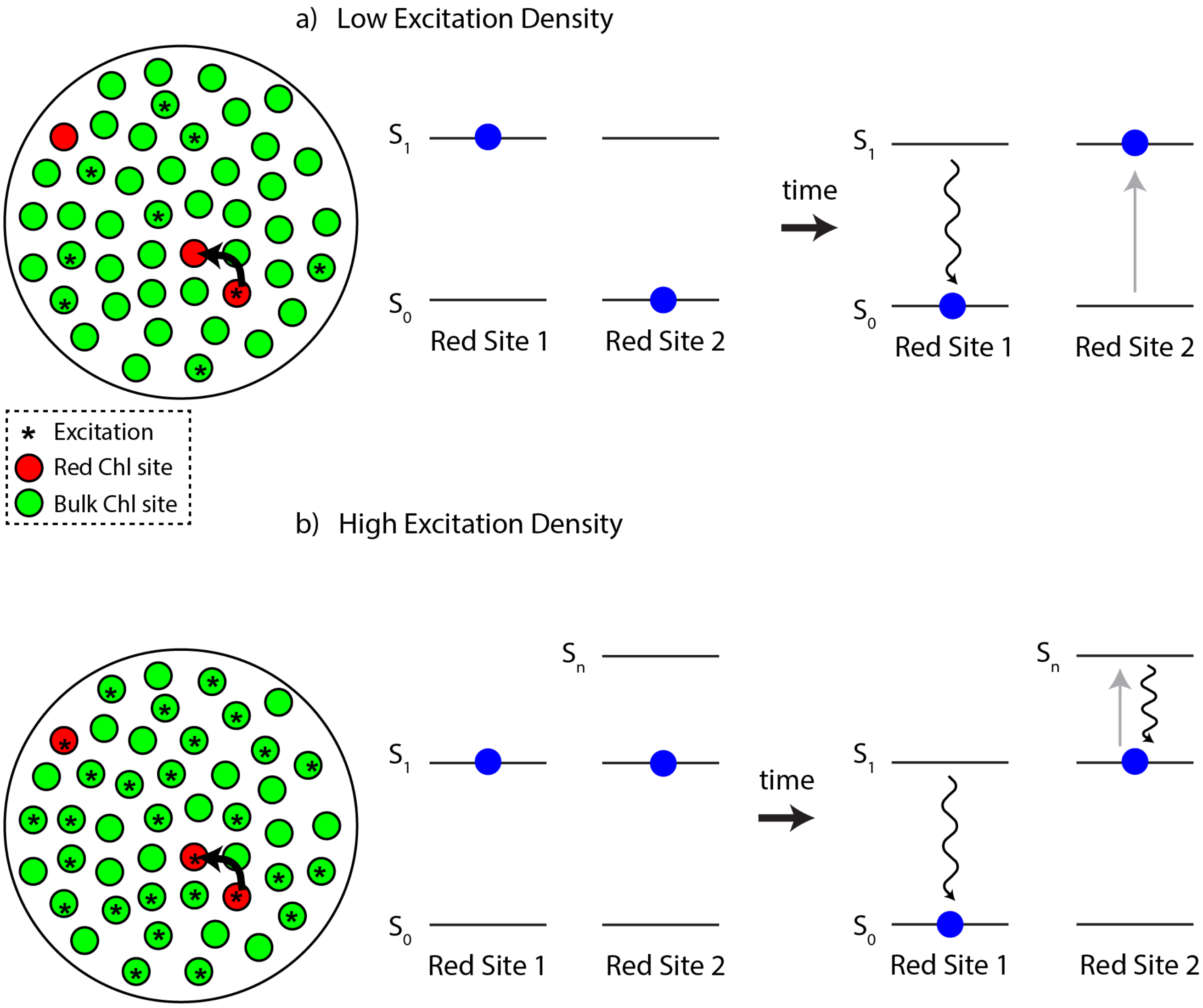

Figure S3. Illustration of energy transfer between energetically connected red Chl sites under a) low excitation density where only one red Chl site is initially excited. Initially, there is a single excitation on red site 1 that transfers to red site 2. b) Under high excitation density, both energetically connected red Chl sites are initially excited. An excitation transfers from red site 1 to red site 2 resulting in singlet-singlet annihilation: an excitation is promoted to a higher excited state (S_n) and then rapidly relaxes back to S_1. The net result is a loss of 1 excitation.

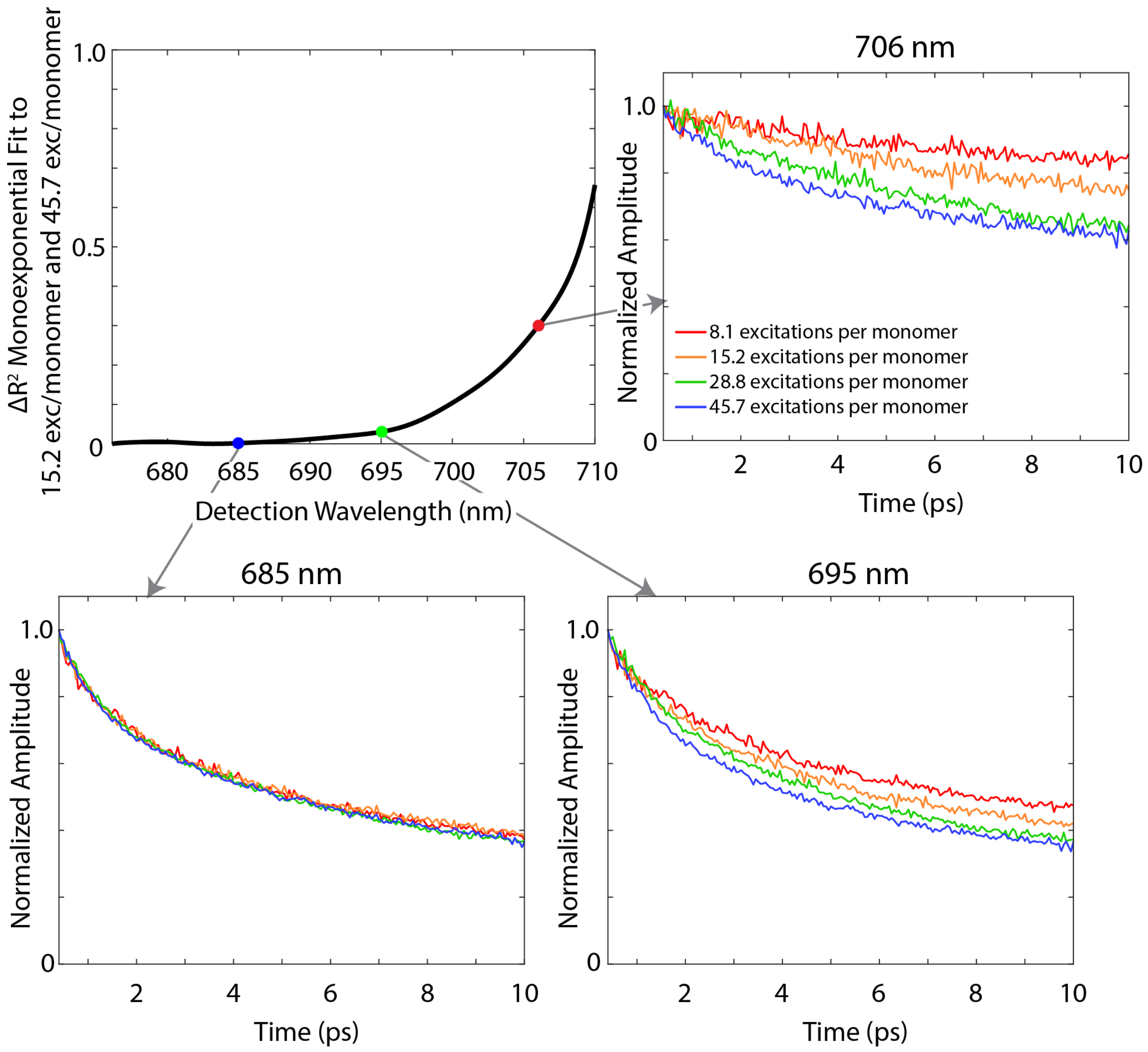

Figure S4. Difference in the R^2 value between a monoexponential decay fit to the waiting time trace from the pump fluence of 39 nJ/pulse (15.2 excitations per monomer, orange waiting time traces) and the calculated R^2 value of the monoexponential fits to 39 nJ/pulse and the waiting time traces from the highest pump fluence of 117 nJ/pulse (45.7 excitations per monomer, blue waiting time traces). A ΔR^2 value of 0 indicates that the monoexponential fits to the data at 39 nJ/pulse and 117 nJ/pulse equally well. At longer wavelengths, ΔR^2 increases indicating that the monoexponential fit to 39 nJ/pulse is a poor fit to the data at 117 nJ/pulse.

FRET calculations

We used the site energies of Yin et al. using INDO calculations on PSI of *T. elongatus* and couplings calculated by Akhtar *et al*. from the *Synechocystis* PSI structure.[5, 6] For calculating the dipole moment of the coupled trimer or dimers, we first calculate the dipole moment directions of the individual chlorophylls. Dipole moment orientations of the coupled dimer or trimer were calculated from the *U* matrix of the diagonalization procedure of the dipole moments of the individual chlorophylls. The eigenvector for the lowest diagonal eigenvalue was used since the dimers are J-aggregates.[7] The new resulting dipole moment was placed at the center of the Mg-Mg distance. FRET rates were calculated using Equation S1 in accordance with work by Sheng *et al*. for Chl *a* in PSII.[8]

$$k_{FRET} = \frac{CK^2}{n^4 R^6} \qquad [S1]$$

We use their reported overlap value, $C = 32.26$.[8] The refractive index n is used as 1.55 and R is the distance between the average position of chlorophyll Mg atoms of the chlorophylls.[9] The obtained distances parameters and FRET rates are summarized in Table S1.

Transfer pairs	K^2	Distance (Å)	Rate^{-1} (ps)
A31/A32/B7 ↔ B37/B38	0.436	19.556	16
A31/A32/B7 ↔ P_{700}	0.998	28.870	73
B37/B38 ↔ P_{700}	0.0049	35.091	253087
B31/B32 ↔ P_{700}	0.5625	44.000	587
B31/B32 ↔ A31/A32/B7	0.1567	69.314	571451
B31/B32 ↔B37/B38	0.562	66.198	33704

Table S1. Orientation factors (K^2), distances, and corresponding time constants from calculated FRET rates.

Calculation of Excitation Densities

Excitation densities of 8.1 – 45.7 excitations/monomer were calculated from the spectrally resolved laser excitation spectrum and linear absorbance spectrum. For transient absorption measurements, the pump pulse was focused to a 0.0050 mm^2 spot through a 500 μm sample cell.

At the lowest pump fluence, the power was measured to be 103 μW which corresponds to 21 nJ/pulse with a laser repetition rate of 5 kHz. This pump fluence was multiplied by the measured laser spectrum shown in **Figure S5** and divided by the spot size of 0.0050 mm^2 to determine the fluence at each wavelength, $I_0(\lambda)$, in units of photons pulse^{-1} cm^{-2}. The spectrally resolved number of photons absorbed per cm^2 were calculated from Equation S2, where $A(\lambda)$ is the absorbance in the 500 μm sample cell. The spectrally resolved number of photons absorbed for the lowest pump fluence, 21 nJ/pulse, is shown in **Figure S5**.

$$Photons\ absorbed\ (\lambda) = I_0(\lambda) - \frac{I_0(\lambda)}{10^{A\,(\lambda)}} \qquad [S2]$$

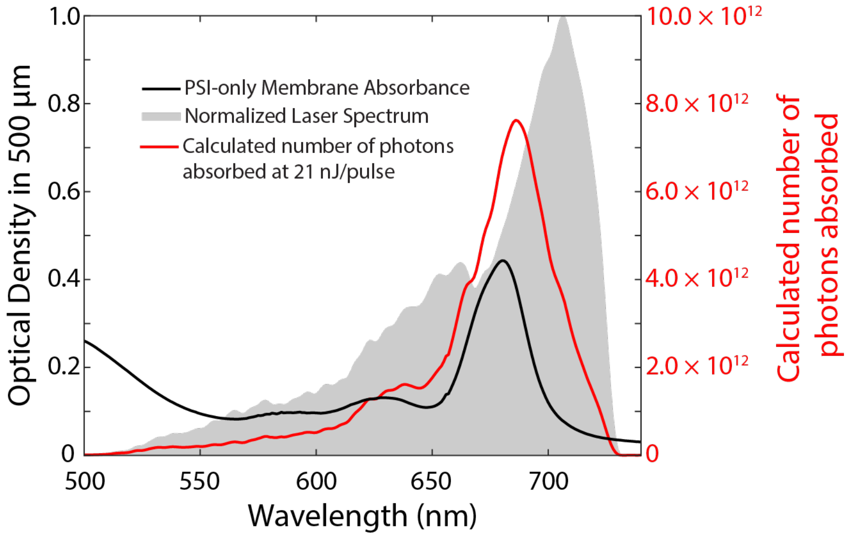

Figure S5. Spectrally resolved calculated number of photons absorbed (red) at the lowest pump fluence of 21 nJ/pulse. Absorbance of PSI-only membranes in a 500 μm sample cell (black). Normalized laser excitation spectrum (shaded gray).

Müh and Zouni determined the molar absorptivity, ε, of protein-bound Chl a in PSI trimers at 680 nm to be 5.7 × 10^4 M^{-1} cm^{-1}.[10] The molar concentration, C, of Chl a in the sample cell with path length $l = 0.05\ cm$ was calculated to be 155.4 µM Chl a from the measured PSI absorbance at 680 nm using Beer-Lambert's law (Equation S3).

$$C = \frac{A\,(680\ nm)}{\varepsilon\, l} \quad [S3]$$

The molar concentration of Chl a was divided by 95, the number of Chl a pigments per PSI monomer, yielding 1.6355 µM PSI monomers. The molar concentration of PSI monomers was then converted to the number of complexes per square centimeter of the sample cell surface by multiplying by the path length, l. The fraction of PSI monomers excited was calculated by dividing the spectrally integrated number of photons absorbed from Equation S2 by the number of PSI complexes per cm^2. Fractions of PSI monomers excited correspond to excitation densities of 8.1, 15.2, 28.8, and 45.7 excitations/monomer, respectively.

Diffusive Random-Walk Model of Energy Transfer at Red Chls

Our fluence-dependent TA results at 706 nm (**Figure 2**) clearly show excitation-density dependent dynamics on a sub 10 ps timescale. While low-temperature hole burning and single-molecule studies support that the red Chls are energetically connected on such fast timescales,[11, 12] our calculated FRET rates for transfer between identified proximal red sites does not fit with our experimental results. In order to understand the observed dynamics from our experiments in the context of existing structural and dynamic data, we test a diffusion-limited random-walk model of energy transfer between red Chl sites using picosecond timescale hopping between red Chl sites.

We construct our model to output only exciton dynamics at and near the red Chls in order to confirm that energetically connected red Chls in trimeric PSI monomers is consistent with the

observed singlet-singlet annihilation. Exciton migration and trapping in PSI lies in an intermediate regime between trap-limited and diffusion-limited kinetics (also known as transfer-to-trap-limited), the former limited by the efficiency of a reversible trap and the latter limited only by the diffusion rate to reach an irreversible trap.[13-17] It is likely that energy transfer from the bulk to P_{700} is a trap- limited process, while uphill energy transfer from red Chls to P_{700} is closer to a diffusion-limited process.[15, 18] At present, we do not model exciton migration through the extensive network of bulk Chls and therefore consider the kinetics to be diffusion-limited.

Our model considers PSI monomers from trimeric and monomeric PSI. Recent work by Beckova *et al.* reports the ratio of PSI monomers to trimers in the Δ*psbB* mutant.[19] We use a ratio of 0.6 monomers in trimers to 0.4 lone monomers in our model. Each modeled monomer contains 15 sites. The trimeric PSI monomer contains three sites corresponding to the red Chl sites (distal site B31/B32; proximal sites A32/B7 and B37/B38). The bulk Chl sites included represent bulk Chl pigments (or dimers) that are physically close to each red Chl site and can transfer absorbed excitations to their associated red Chl site on an ultrafast timescale. Modeled lone PSI monomers only have two sites corresponding to red Chl sites as one of the proximal sites blue shifts when it is not a part of a PSI trimer such that it is no longer considered a red Chl site.[18, 20, 21] The remaining 12 or 13 sites are bulk Chls and each red Chl site is associated with 4 bulk Chl sites. **Figure S6** shows the sites used to model monomeric and trimeric PSIs. As it is our intention only to reproduce our experimental TA traces at 706 nm (red Chl signal), explicitly considering all 95 Chls in PSI falls outside the scope of this work.

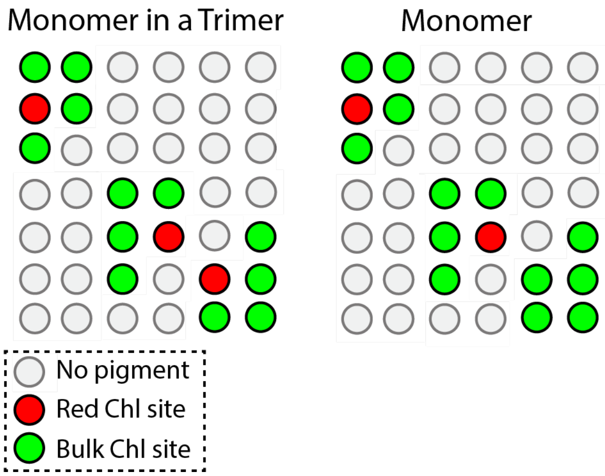

Figure S6. Matrices used in random walk model of trimeric PSI monomer (Left) and lone PSI monomer (Right). Both forms of the monomer contain a distal Red Chl (red circle, column 1, row 2). The trimeric monomer contains a pair of energetically connected proximal red Chls (red circles, column 4, row 6 and column 5, row 7). The monomeric PSI monomer contains a single proximal red Chl (red circle, column 4, row 6). In the model, excitations can hop between nearest neighbor pigments with probabilities determined by the time constants of the transfer process (Table S2) relative to the duration of the time step.

We randomly populate the modeled Chl sites with initial excitations based on the experimental laser fluences. Based on the absorption spectrum of our sample and the experimental laser spectrum, approximately 5% of absorbed photons will directly excite the red Chl sites. Depending on the oligomerization state of PSI, we model 12 or 13 bulk sites which accounts for between 12 and 26 individual Chl pigments (due to the possibility that the bulk Chl sites may be dimeric or monomeric). Of the 95 Chls in PSI, 87 are bulk Chls; therefore, our 12 (or 13) bulk sites represent between 13.8% (14.9%) and 27.6% (29.9%) of the total bulk Chl pigments. In our model, we set absorption by the included bulk Chl sites at 15% of the total absorbed excitations. As a result, 20% of absorbed photons are included in our model. Thus, our experimental fluences corresponding to 8.1, 15.2, 28.8, and 45.7 excitations per monomer gives us average excitation densities of 1.62, 3.04, 5.76, and 9.14 excitations distributed across the 15 sites in our modeled

monomers. **Figures S7 and S8** show representative samples of eight modeled monomers at T = 0 for the lowest and highest fluences, respectively.

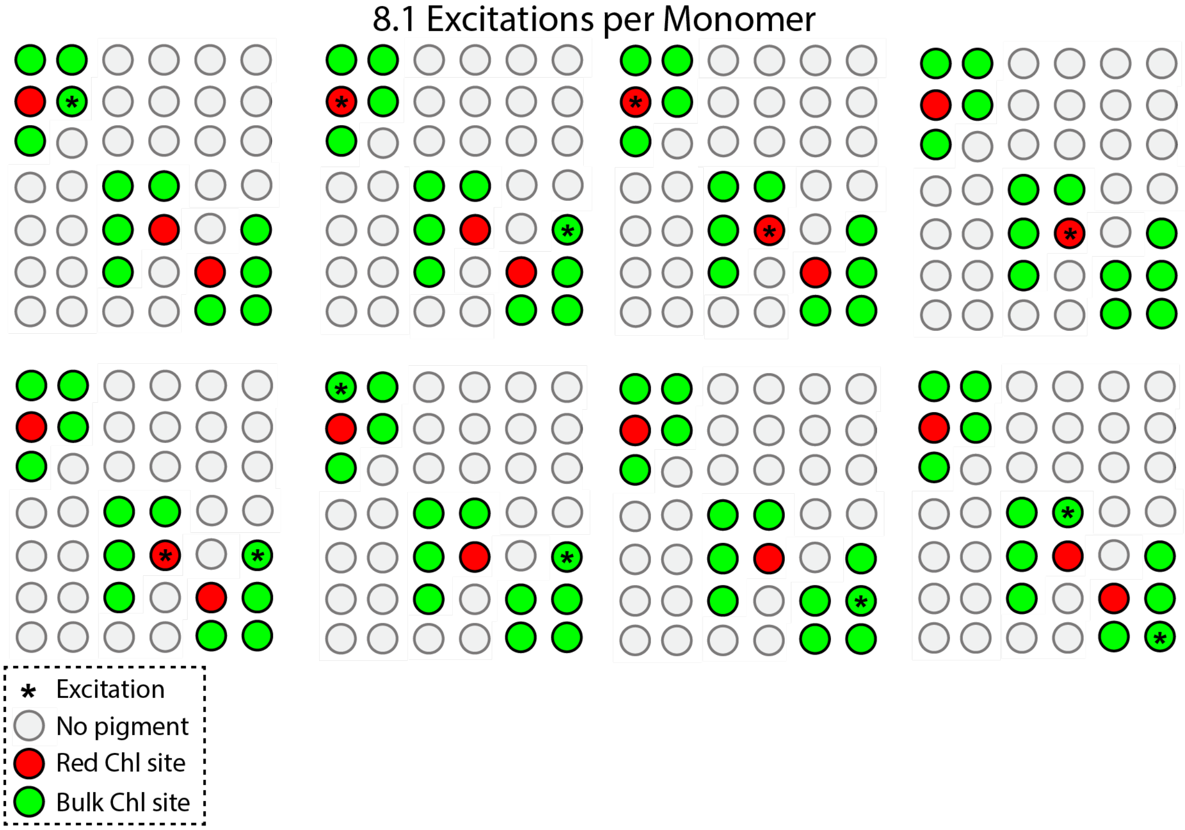

Figure S7. Eight matrices from random walk model for the excitation fluence corresponding to 8.1 excitations per monomer at T = 0. Each matrix is randomly populated with excitations based on the experimental fluence. With 20% of experimental absorbed excitations considered, there is an average of 1.62 excitations per monomer in the model. These matrices represent a mixture of trimeric and non-trimeric PSI monomers (60% of monomers are in trimers) and the non-trimeric monomers lack one of the proximal red Chl sites.

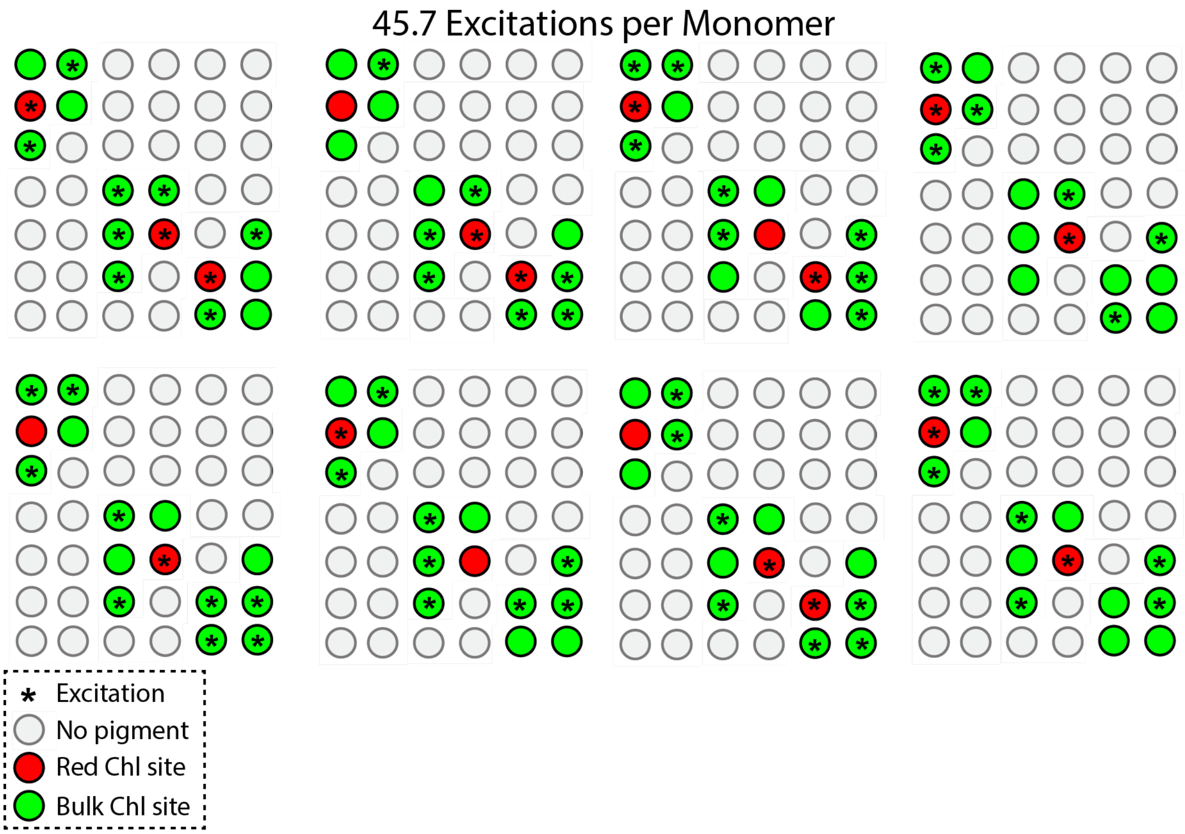

Figure S8. Matrices from random walk model for the excitation fluence corresponding to 45.7 excitations per monomer at T = 0. Each matrix is randomly populated with excitations based on the experimental fluence. With 20% of experimental absorbed excitations considered, there is an average of 9.14 excitations per monomer in the model. These matrices represent a mixture of trimeric and non-trimeric PSI monomers (60% of monomers are in trimers) and the non-trimeric monomers lack one of the proximal red Chl sites.

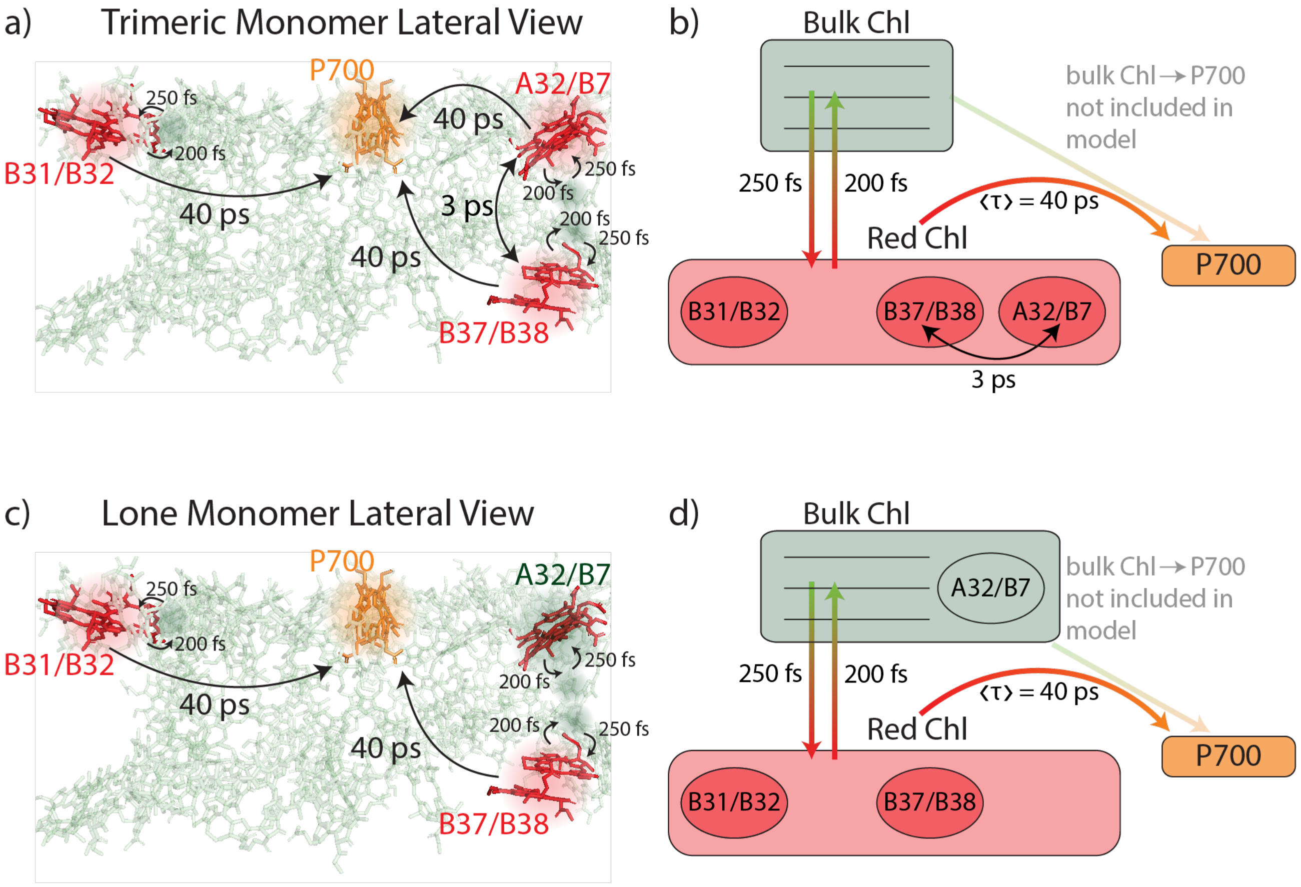

Figure S9. Assigned Chl sites and hopping times used in the model of (a-b) trimeric PSI monomers and (c-d) monomeric PSI monomers giving the best fit to experimental data (Figure 3). Downhill energy transfer is allowed from bulk Chls (green) in close proximity to red Chl sites (red) with a 250 fs time constant. Uphill energy transfer is allowed from bulk to red Chl sites with a 200 fs time constant. Excitations are allowed to transfer from a red Chl site to P_{700} (orange) with a 40 ps time constant. In trimeric PSI monomers (a-b), excitations are allowed to transfer between proximal red Chl sites within the same monomer with a 3 ps time constant. In monomeric PSI monomers (c-d), A32/B7 is treated as a bulk Chl site. a, c) Lateral zoomed-in view of a PSI monomer. Chl locations are shown from the crystal structure of the PSI trimer (PDB ID: 5OY0[22]) using the red Chl assignments made by Akhtar *et al.*[21] b, d) A cartoon visualization of the kinetic model.

Figure S9 illustrates the allowed energy transfer pathways and assigned time constants built into the model of trimeric monomers and the analogous pathways and time constants for lone monomers. We only consider processes that will directly influence the dynamics at the red Chls. In modeled trimeric PSI monomers, exciton hopping can occur between the two proximal red Chl sites with a hopping time on the picosecond timescale. This hopping timescale is implied by our

experimental TA results, but is not suggestive of a FRET-like transfer mechanism given the calculated FRET hopping times in **Table S1**. Excitations can transfer from bulk Chl sites to the associated red Chls on a 250 fs timescale and back on a 200 fs timescale.[1] Fast transfer from bulk sites effectively increases the excitation density at the red Chls and has been suggested by previous single molecule studies.[23] During each 10 fs time step, excitations are allowed to hop to a neighboring site (i.e., bulk Chl to red Chl, red Chl to bulk Chl, red Chl to red Chl), or migrate to P_{700}, with a probability determined by the time constants of these processes (**Table S2**) relative to the duration of the step. Back transfer between the red Chls and their associated bulk Chls is weighted relative to forward transfer based on the Boltzmann probabilities of transfer in each direction. This uphill energy transfer is entropically favorable due to the large number of bulk Chl sites.[24] Previous reports support that the transfer rate to P_{700} is not strongly affected by a closed (oxidized) versus open (reduced) RC,[16, 21, 25-27] therefore, we use a single value of 40 ps for the average transfer time from the red Chls to P_{700}, which provides the best fit to the experimental data (**Figure 3**). We include a long-lived timescale component of 2 ns, as the experimental TA traces (**Figure 2**) clearly show that excitations persist at the red Chls even at the highest excitation density well-beyond the 10 ps window of the measurement. The long-lived component cannot be precisely determined because it is two orders of magnitude longer than the time window of our measurement. **Figure S12** shows a comparison of the modeled time traces using 2 ns and 5 ns time constants, timescales suggested by other electronic spectroscopy studies of PSI.[1, 6] The modeled decays using a 2 ns or 5 ns long-lived decay component are comparable.

Transfer Process	Time constant
Bulk Chls to red Chls (distal or proximal)	250 fs
Red Chls to bulk Chls (distal or proximal)	200 fs
Proximal red Chl to proximal red Chl	3 ps
Red Chls (distal or proximal) to P_{700}	40 ps
Red Chls long-lived decay	2 ns

Table S2. Energy transfer times used in diffusive random walk model of energy transfer at the red Chls as shown in **Figure 3 (also reproduced in Figures S10b, S11b, and S12a)**.

Annihilation occurs when two or more excitations occupy the same site due to exciton migration. In the model, annihilation reduces the number of excitations at a single site from two to one. A similar model was used by Dahlberg *et al.* to understand the membrane architecture in *Rhodobacter sphaeroides* cells.[28] We use our model to generate random walk trajectories that that can be compared to the experimental data traces at 706 nm (**Figures S10-S12**).

This random walk model can reproduce the fluence-dependence of the 706 nm signal in our TA data after an optimization of the hopping time between proximal red Chl sites, with the caveat that we do not have a complete understanding of the physical basis for the picosecond hopping between proximal red Chls. A red Chl-red Chl hopping time of 3 ps yields the best fit to the experimental data (**Figure 3**) but disagrees with a FRET-like hopping mechanism. **Figure S13** shows the model output for the bulk Chls. While fluence-dependent dynamics are present in the first 800 fs of the model, the bulk Chls show no fluence-dependent dynamics after the initial 800 fs, in line with our experimental results (**Figure 2**). The model results presented in **Figure S11-S13** represent 5000 iterations of our 10 ps random walk (1000, 10-fs time steps per iteration). All calculations were performed in Jupyter Notebook and code is available on Github.[29]

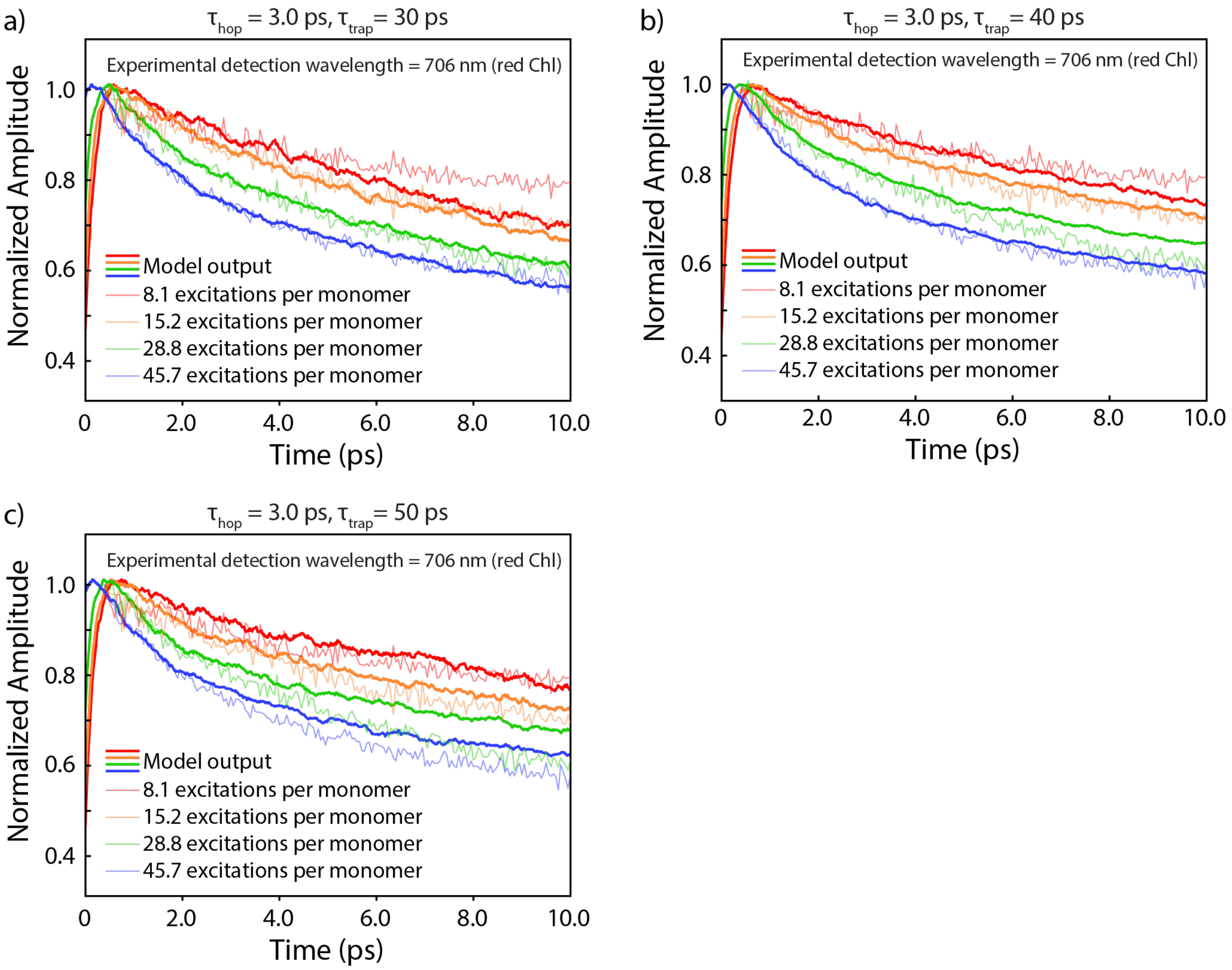

Figure S10. Overlay of modeled red Chl waiting time traces (bold lines) using trapping times of a) 30 ps, b) 40 ps (this is a reproduction of **Figure 3**), and c) 50 ps at each excitation density with experimental waiting time traces from the detection wavelength 706 nm (semi-transparent lines). All other time constants are detailed in Table S2. Model results for trapping times of 30 ps and 50 ps are the average of 2000 iterations; results for the trapping time of 40 ps are the average of 5000 iterations and all are individually normalized to the maximum.

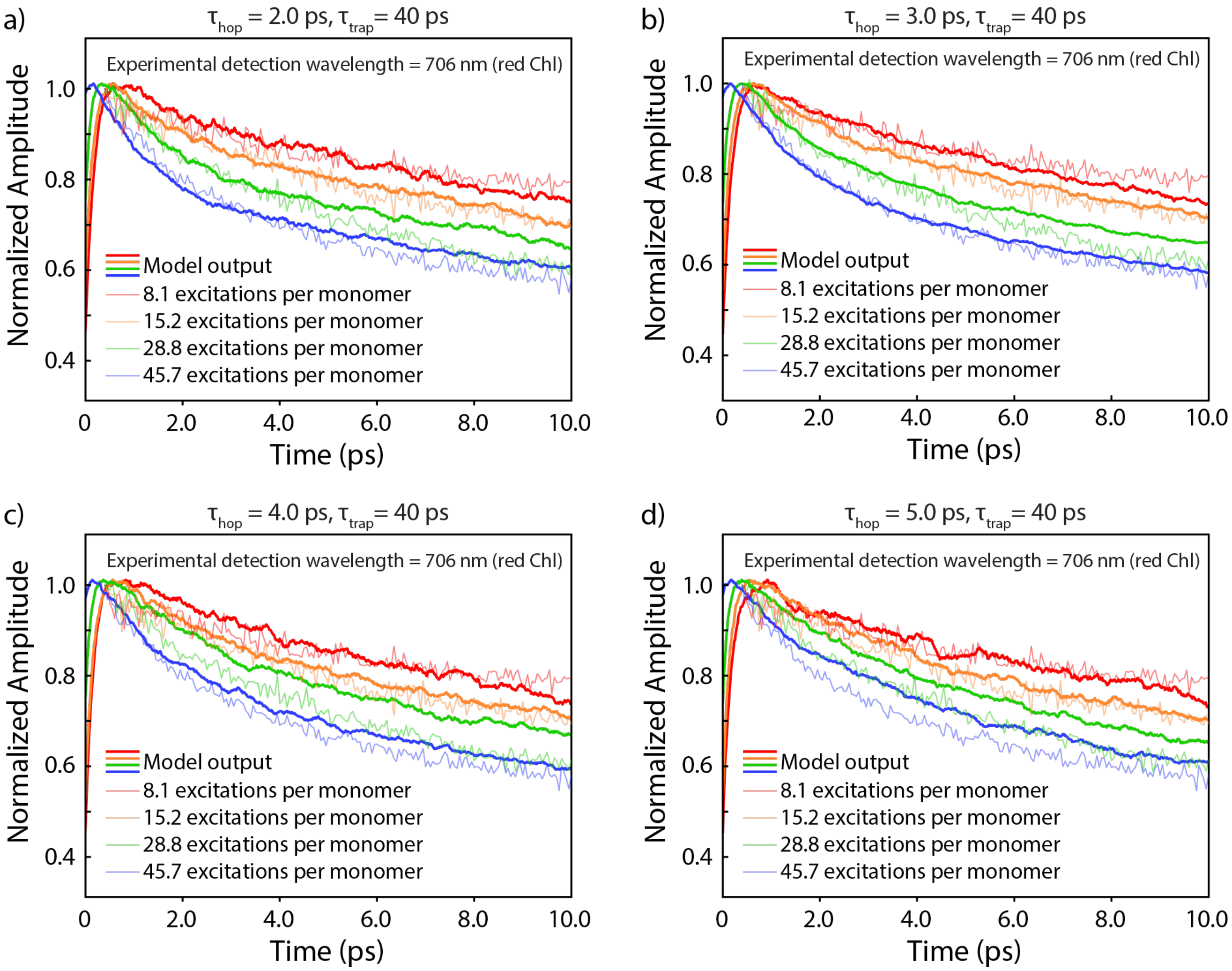

Figure S11. Overlay of modeled red Chl waiting time traces (bold lines) using red Chl-red Chl hopping times of a) 2.0 ps, b) 3.0 ps (this is a reproduction of **Figure 3**), c) 4.0 ps, and d) 5.0 ps at each excitation density with experimental waiting time traces from the detection wavelength 706 nm (semi-transparent lines). All other time constants are detailed in Table S2. Model results are the average of 5000 iterations and are individually normalized to the maximum.

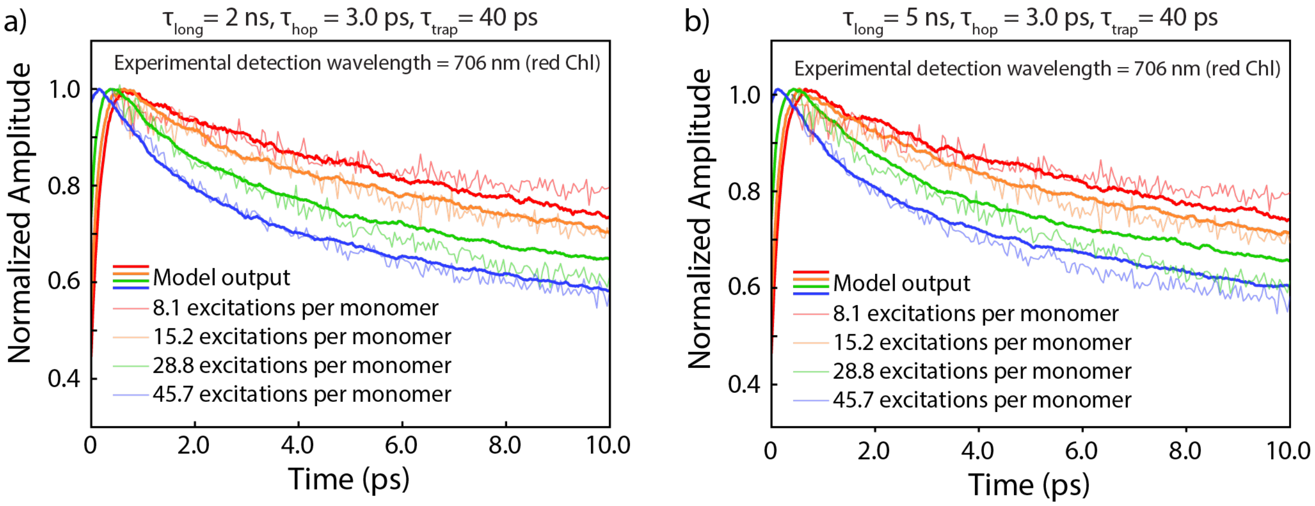

Figure S12. Overlay of modeled red Chl waiting time traces (bold lines) using a long-lived decay component of a) 2.0 ns (this is a reproduction of **Figure 3**), and b) 5.0 ns at each excitation density with experimental waiting time traces from the detection wavelength 706 nm (semi-transparent lines). All other time constants are detailed in Table S2. Model results are the average of 5000 iterations and are individually normalized to the maximum.

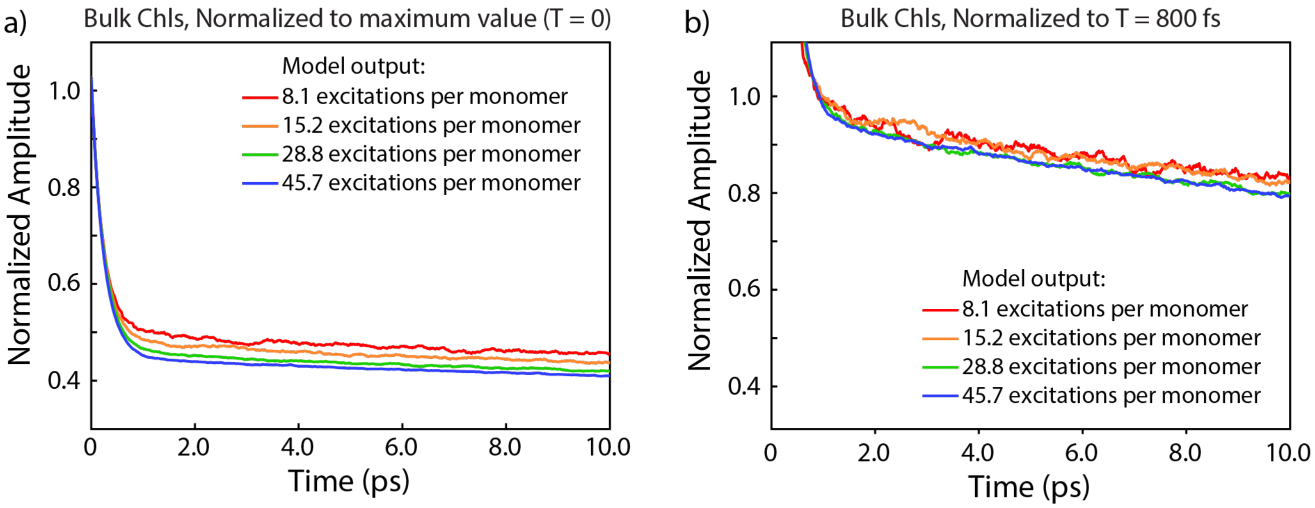

Figure S13. Modeled bulk Chl waiting time traces using the time constants detailed in Table S2. Model results are the average of 5000 iterations for each fluence and are a) individually normalized to the maximum (T = 0) and b) individually normalized at T = 800 fs.

References

1. Lee, Y.; Gorka, M.; Golbeck, J. H.; Anna, J. M., Ultrafast Energy Transfer Involving the Red Chlorophylls of Cyanobacterial Photosystem I Probed through Two-Dimensional Electronic Spectroscopy. *J. Am. Chem. Soc.* **2018,** *140*, 11631-11638.
2. van Stokkum, I. H.; Larsen, D. S.; van Grondelle, R., Global and Target Analysis of Time-resolved Spectra. *Biochim. Biophys. Acta* **2004,** *1657*, 82-104. doi:10.1016/j.bbabio.2004.04.011
3. Ruckebusch, C.; Sliwa, M.; Pernot, P.; de Juan, A.; Tauler, R., Comprehensive Data Analysis of Femtosecond Transient Absorption Spectra: A Review. *J. of Photochem. Photobiol. C: Photochem. Rev.* **2012,** *13*, 1-27. doi:10.1016/j.jphotochemrev.2011.10.002
4. Virtanen, P.; Gommers, R.; Oliphant, T. E.; Haberland, M.; Reddy, T.; Cournapeau, D.; Burovski, E.; Peterson, P.; Weckesser, W.; Bright, J.; van der Walt, S. J.; Brett, M.; Wilson, J.; Millman, K. J.; Mayorov, N.; Nelson, A. R. J.; Jones, E.; Kern, R.; Larson, E.; Carey, C. J.; Polat, I.; Feng, Y.; Moore, E. W.; VanderPlas, J.; Laxalde, D.; Perktold, J.; Cimrman, R.; Henriksen, I.; Quintero, E. A.; Harris, C. R.; Archibald, A. M.; Ribeiro, A. H.; Pedregosa, F.; van Mulbregt, P.; SciPy, C., SciPy 1.0: Fundamental Algorithms for Scientific Computing in Python. *Nat. Methods* **2020,** *17*, 261-272. doi:10.1038/s41592-019-0686-2
5. Yin, S.; Dahlbom, M. G.; Canfield, P. J.; Hush, N. S.; Kobayashi, R.; Reimers, J. R., Assignment of the Qy Absorption Spectrum of Photosystem-I from *Thermosynechococcus elongatus* Based on CAM-B3LYP Calculations at the PW91-Optimized Protein Structure. *J. Phys. Chem. B* **2007,** *111*, 9923-9930.
6. Akhtar, P.; Caspy, I.; Nowakowski, P. J.; Malavath, T.; Nelson, N.; Tan, H. S.; Lambrev, P. H., Two-Dimensional Electronic Spectroscopy of a Minimal Photosystem I Complex Reveals the Rate of Primary Charge Separation. *J. Am. Chem. Soc.* **2021,** *143*, 14601-14612. doi:10.1021/jacs.1c05010
7. Hestand, N. J.; Spano, F. C., Expanded Theory of H- and J-Molecular Aggregates: The Effects of Vibronic Coupling and Intermolecular Charge Transfer. *Chem. Rev.* **2018,** *118*, 7069-7163. doi:10.1021/acs.chemrev.7b00581
8. Sheng, X.; Watanabe, A.; Li, A.; Kim, E.; Song, C.; Murata, K.; Song, D.; Minagawa, J.; Liu, Z., Structural Insight into Light Harvesting for Photosystem II in Green Algae. *Nat. Plants* **2019,** *5*, 1320-1330. doi:10.1038/s41477-019-0543-4
9. Gradinaru, C. C.; Ozdemir, S.; Gulen, D.; van Stokkum, I. H.; van Grondelle, R.; van Amerongen, H., The Flow of Excitation Energy in LHCII Monomers: Implications for the Structural Model of the Major Plant Antenna. *Biophys. J.* **1998,** *75*, 3064-3077. doi:10.1016/S0006-3495(98)77747-1
10. Müh, F.; Zouni, A., Extinction Coefficients and Critical Solubilisation Concentrations of Photosystems I and II from *Thermosynechococcus elongatus*. *Biochim. Biophys. Acta* **2005,** *1708*, 219-228. doi:10.1016/j.bbabio.2005.03.005
11. Hsin, T.-M.; Zazubovich, V.; Hayes, J. M.; Small, G. J., Red Antenna States of PS I of Cyanobacteria: Stark Effect and Interstate Energy Transfer. *J. Phys. Chem. B* **2004,** *108*, 10515-10521. doi:10.1021/jp049572m
12. Riley, K. J.; Reinot, T.; Jankowiak, R.; Fromme, P.; Zazubovich, V., Red Antenna States of Photosystem I from Cyanobacteria *Synechocystis* PCC 6803 and *Thermosynechococcus elongatus*: Single-Complex Spectroscopy and Spectral Hole-Burning Study. *J. Phys. Chem. B* **2007,** *111*, 286-292.
13. van Grondelle, R.; Dekker, J. P.; Gillbro, T.; Sundstrom, V., Energy Transfer and Trapping in Photosynthesis. *Biochim. Biophys. Acta* **1994,** *1187*, 1-65.
14. van Thor, J. J.; Mullineaux, C. W.; Matthijs, H. C. P.; Hellingwerf, K. J., Light Harvesting and State Transitions in Cyanobacteria. *Bot. Acta* **1998,** *111*, 430-443.

15. Savikhin, S., Ultrafast Optical Spectroscopy of Photosystem I. In *Photosystem I: The Light-Driven Plastocyanin:Ferredoxin Oxidoreductase*, Golbeck, J. H., Ed. Springer: Dordrecht, The Netherlands, 2006; Vol. 24, pp 155-175.
16. Byrdin, M.; Rimke, I.; Schlodder, E.; Stehlik, D.; Roelofs, T. A., Decay Kinetics and Quantum Yields of Fluorescence in Photosystem I from Synechococcus elongatus with P700 in the Reduced and Oxidized State: Are the Kinetics of Excited State Decay Trap-Limited or Transfer-Limited? *Biophys. J.* **2000,** *79*, 992-1007. doi:10.1016/S0006-3495(00)76353-3
17. Gobets, B.; Dekker, J. P.; van Grondelle, R., Transfer-to-the-Trap Limited Model of Energy Transfer in Photosystem 1. In *Photosynthesis: Mechanisms and Effects*, Garab, G., Ed. Springer: Dordrecht, 1998; pp 503-508.
18. Karapetyan, N. V.; Holzwarth, A. R.; Rogner, M., The Photosystem I Trimer of Cyanobacteria: Molecular Organization, Excitation Dynamics and Physiological Significance. *FEBS Lett.* **1999,** *460*, 395-400.
19. Beckova, M.; Sobotka, R.; Komenda, J., Photosystem II Antenna Modules CP43 and CP47 Do Not Form a Stable 'No Reaction Centre Complex' in the Cyanobacterium *Synechocystis* sp. PCC 6803. *Photosynth. Res.* **2022,** *152*, 363-371. doi:10.1007/s11120-022-00896-w
20. Hayes, J. M.; Matsuzaki, S.; Ratsep, M.; Small, G. J., Red Chlorophyll *a* Antenna States of Photosystem I of the Cyanobacterium *Synechocystis* sp. PCC 6803. *J. Phys. Chem. B* **2000,** *104*, 5625-5633.
21. Akhtar, P.; Biswas, A.; Kovacs, L.; Nelson, N.; Lambrev, P. H., Excitation Energy Transfer Kinetics of Trimeric, Monomeric and Subunit-Depleted Photosystem I from *Synechocystis* PCC 6803. *Biochem. J.* **2021,** *478*, 1333-1346. doi:10.1042/BCJ20210021
22. Malavath, T.; Caspy, I.; Netzer-El, S. Y.; Klaiman, D.; Nelson, N., Structure and Function of Wild-Type and Subunit-Depleted Photosystem I in *Synechocystis*. *Biochim. Biophys. Acta Bioenerg.* **2018,** *1859*, 645-654. doi:10.1016/j.bbabio.2018.02.002
23. Brecht, M.; Radics, V.; Nieder, J. B.; Bittl, R., Protein Dynamics-Induced Variation of Excitation Energy Transfer Pathways. *Proc. Natl. Acad. Sci. USA* **2009,** *106*, 11857-11861. doi:10.1073/pnas.0903586106
24. Melkozernov, A. N.; Lin, S.; Blankenship, R. E.; Valkunas, L., Spectral Inhomogeneity of Photosystem I and Its Influence on Excitation Equilibration and Trapping in the Cyanobacterium *Synechocystis sp.* PCC6803 at 77 K. *Biophys. J.* **2001,** *81*, 1144-1154. doi:10.1016/S0006-3495(01)75771-2
25. Karapetyan, N. V.; Schlodder, E.; van Grondelle, R.; Dekker, J. P., The Long Wavelength Chlorophylls of Photosystem I. In *Photosystem I: The Light-Driven Plastocyanin:Ferredoxin Oxidoreductase*, Golbeck, J. H., Ed. Springer: Dordrecht, The Netherlands, 2006; Vol. 24, pp 177-192.
26. Shibata, Y.; Yamagishi, A.; Kawamoto, S.; Noji, T.; Itoh, S., Kinetically Distinct Three Red Chlorophylls in Photosystem I of *Thermosynechococcus elongatus* Revealed by Femtosecond Time-Resolved Fluorescence Spectroscopy at 15 K. *J. Phys. Chem. B* **2010,** *114*, 2954-2963.
27. Schlodder, E.; Hussels, M.; Cetin, M.; Karapetyan, N. V.; Brecht, M., Fluorescence of the Various Red Antenna States in Photosystem I Complexes from Cyanobacteria is Affected Differently by the Redox State of P700. *Biochim. Biophys. Acta* **2011,** *1807*, 1423-1431. doi:10.1016/j.bbabio.2011.06.018
28. Dahlberg, P. D.; Ting, P.-C.; Massey, S. C.; Allodi, M. A.; Martin, E. C.; Hunter, C. N.; Engel, G. S., Mapping the Ultrafast Flow of Harvested Solar Energy in Living Photosynthetic Cells. *Nat. Commun.* **2017,** *8*, 988-994. doi:10.1038/s41467-017-01124-z
29. Sohail, S. H. SohailLab/PSI_RedChl_May2024. https://doi.org/10.5281/zenodo.11392154.
